# CD19 CAR T cell therapy BY19 for pediatric and adult patients with relapsed or refractory B cell neoplasms in Belarus: Phase 1 trial

**DOI:** 10.1016/j.omton.2025.201081

**Published:** 2025-11-01

**Authors:** Mikalai Katsin, Dmitri Dormeshkin, Alexandr Migas, Olga Karas, Tatsiana Shman, Yuliya Serada, Yauheniya Khalankova, Hanna Klych, Dzmitry Lutskovich, Alena Lukoika, Alexander Meleshko

**Affiliations:** 1 Department of Hematology, Vitebsk Regional Clinical Cancer Centre, 210603 Vitebsk, Belarus; 2 Laboratory of Molecular Diagnostics and Biotechnology, Institute of Bioorganic Chemistry of the National Academy of Sciences of Belarus, 220084 Minsk, Belarus; 3 Laboratory of Genetic Biotechnologies, Belarusian Research Center for Pediatric Oncology, Hematology and Immunology, 223053 Minsk, Belarus

**Keywords:** MT: Regular Issue, CD19 CAR T cells, decitabine, B cell acute lymphoblastic leukemia, B-ALL, diffuse large B cell lymphoma, DLBCL, non-Hodgkin lymphoma, NHL

## Abstract

Access to chimeric antigen receptor (CAR) T cell therapy remains limited in many developing countries. We conducted a single-arm, open-label, phase 1 trial (NCT05333302) at two Belarusian centers, evaluating an in-house manufactured CD19 CAR T cell product (BY19) in pediatric and adult patients with relapsed/refractory B cell malignancies. Lymphodepletion included fludarabine/cyclophosphamide, with or without decitabine. Twenty-three patients received therapy: seventeen B cell acute lymphoblastic leukemia, one chronic lymphocytic leukemia, and five non-Hodgkin lymphomas. Cytokine release syndrome (CRS) occurred in 67% of infusions, mostly grade 1–2, with severe CRS in 19%. Immune effector cell-associated neurotoxicity occurred in 44%, with severe cases in 18.5%. The overall response rate was 80% (16/20 evaluable), with complete remission achieved in 75% at day 28. Median progression-free survival (PFS) was 23 months; 12-month PFS was 83.3% in lymphoma and 48.3% in B cell acute lymphoblastic leukemia (B-ALL). Higher C_max_ levels tended to correlate with better response rates (*p* = 0.0416); however, no clear advantage in PFS was observed. BY19’s safety and efficacy profiles were comparable to approved CD19 CAR T cell products. This study underscores the translational potential of localized CAR T cell manufacturing to expand global access to advanced immunotherapies, especially in middle-income countries. Decitabine-containing lymphodepletion showed potential benefit and warrants further study.

## Graphical abstract

**Figure:**
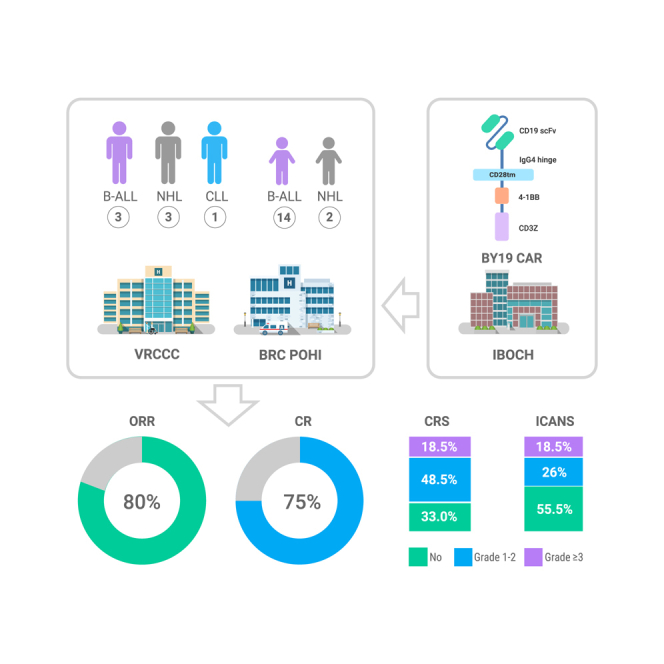

## Teaser

This first-in-region phase 1 trial demonstrates the safety, efficacy, and feasibility of in-house manufactured CD19 CAR T cells (BY19) for relapsed/refractory B cell malignancies in Belarus. Locally produced under GMP-adapted conditions, BY19 achieved 75% complete remission with manageable toxicity, highlighting a cost-effective model for expanding CAR T cell access in middle-income countries.

## Introduction

Approximately 10% of children and young adults treated for upfront B cell acute lymphoblastic leukemia (B-ALL) relapse or become refractory. The 5-year overall survival (OS) rate following first relapse in this population is approximately 50%.^1^^,^^2^ This rate drops to below 40% when patients have minimal residual disease (MRD)-positive status at the end of consolidation therapy.^3^ In adults, the prognosis is considerably worse, with fewer than 10% of patients achieving long-term survival.^4^

Diffuse large B cell lymphoma (DLBCL) is the most common subtype of non-Hodgkin lymphoma (NHL). While first-line treatment yields 5-year survival rates between 60% and 70%, up to 50% of patients either become refractory or relapse after initial therapy. For refractory DLBCL, the objective response rate (ORR) to subsequent treatments is approximately 26%, with only about 7% achieving complete response (CR), and the median OS remains around 6.3 months.^5^ There has been a pressing need for novel therapeutic approaches to improve outcomes in these patient populations.

CD19 chimeric antigen receptor T cell (CAR T) therapy has revolutionized treatment of B cell malignancies. Five CD19 CAR T products have received Food and Drug Administration (FDA) approval, demonstrating CR rates of 60%–93% in B-ALL and 40%–50% in DLBCL, whereas in patients with chronic lymphocytic leukemia (CLL), CR rates of 15%–30% have been observed.^6^^,^^7^^,^^8^^,^^9^^,^^10^

However, widespread clinical adoption is limited by high manufacturing costs, centralized production logistics, lengthy turnaround times, and complex regulatory requirements—factors that collectively restrict access, particularly in low- and middle-income countries.

**Figure 1:**
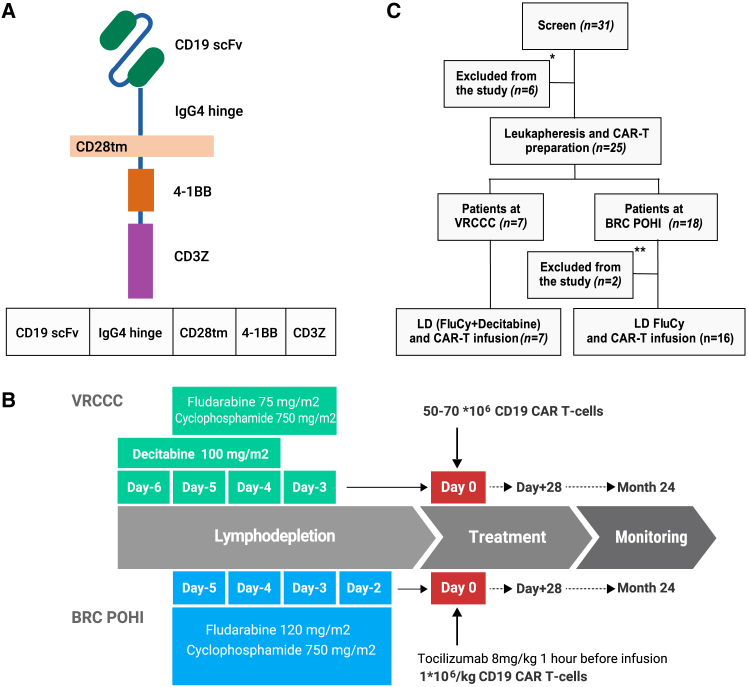
Study design (A) Anti-CD19 CAR design, CD19 scFv (derived from FMC63 antibody); IgG4, short 12AA hinge; CD28tm, transmembrane domain from CD28; 4-1BB, co-stimulatory domain from 4-1BB; CD3Z, CD3z signaling domain. (B) Design of the phase 1 clinical trial NCT05333302. (C) Flowchart for CD19 CAR T cell clinical trial including cell procurement and treatment.

To address this critical gap, we have developed an anti-CD19 CAR design that incorporates an IgG4 hinge and a 4-1BB cytoplasmic domain for our clinical program targeting patients with B cell neoplasms (Figure 1A). Considering the immunological benefits of decitabine (DAC) observed in enhancing CD19 CAR T cell activity against B-ALL and NHL, we have included DAC in the lymphodepletion regimen for adult patients with B cell neoplasms undergoing CD19 CAR T cell therapy.^11^^,^^12^

Here, we present the results of a phase 1 study (ClinicalTrials.gov identifier: NCT05333302) evaluating CD19 CAR T cells (BY19) in patients with relapsed or refractory B cell malignancies conducted in the Republic of Belarus, demonstrating both the feasibility of manufacturing and preliminary durable responses across different B cell neoplasms.

This study represents the first formal clinical investigation of an academic CAR T cell product in Eastern Europe and provides important translational insight into expanding access to CAR T cell therapies in resource-constrained healthcare systems.

## Results

### BY19 CAR *in vitro* characterization

We constructed two of the most widely used designs of second-generation anti-CD19 CARs, based on anti-CD19 FMC63 single-chain variable fragment (scFv)-binding domain: BY19 CAR, which incorporates an IgG4 hinge, a CD28 transmembrane domain, a 4-1BB cytoplasmic domain, and a CD3ζ signaling domain; and a second 28Z CAR possessing CD28 hinge, transmembrane, and cytoplasmic domains (Table S1). In our hands, BY19 CAR T cells exhibited lower surface expression compared with 28Z CAR T cells (Figures S1A and S1B). Despite reduced surface expression, BY19 CAR T cells demonstrated significantly enhanced activation signaling in both nuclear factor κB and NFAT reporter assays (Figures S1C and S1D). In short-term cytotoxicity assays, both CAR T cell designs exhibited similar levels of cytotoxicity (Figure S2A), although 28Z CAR T cells produce higher levels of interferon-γ (Figure S2B).

To compare the functional persistence and cancer cell killing capacity of BY19 and 28Z CAR T cells, we performed a long-term rechallenge assay. After five rounds of re-exposure to CD19^+^ cancer cell lines (Raji and CII), BY19 CAR T cells were superior in terms of specific cytolytic activity and proliferation (Figures S2C and S2D).

On the basis of preclinical evaluation, BY19 CAR T cells were identified to have potent and antigen-specific anti-leukemia activity and were used in the subsequent clinical study.

### Patient characteristics

**Figure 2:**
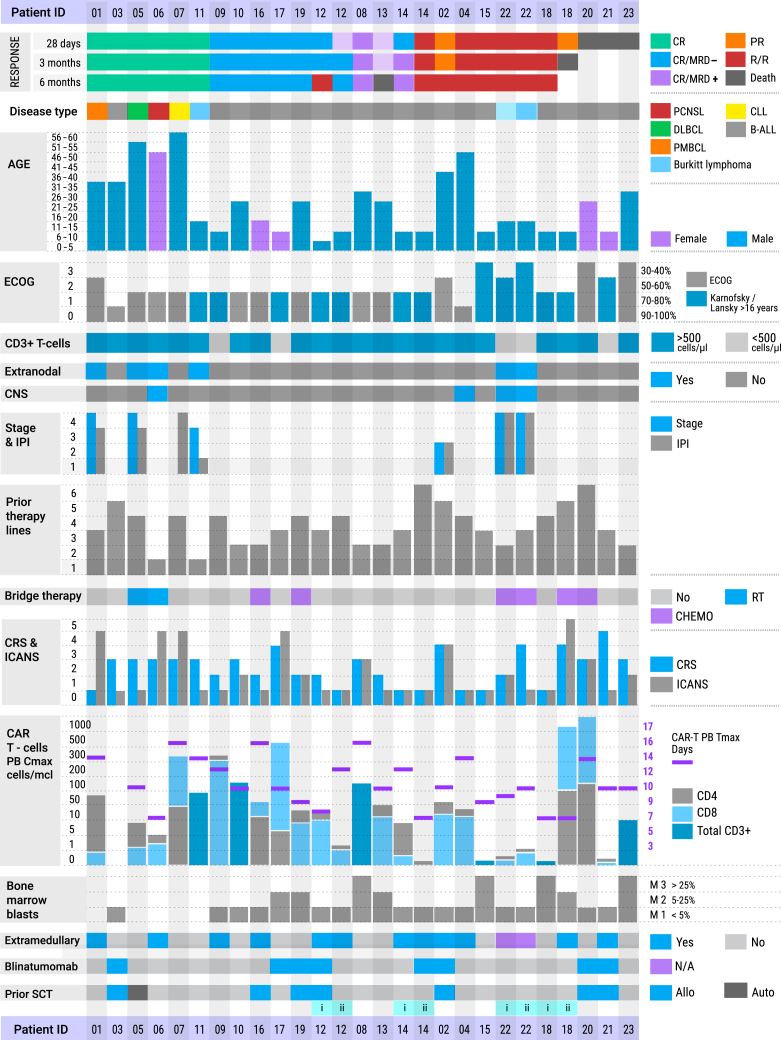
Summary of patient’s characteristics, toxicity, and clinical response data (*n* = 23) DLBCL, diffuse large B cell lymphoma; PCNSL, primary CNS lymphoma; PMBCL, primary mediastinal large B cell lymphoma; CLL, chronic lymphocytic leukemia; B-ALL, B cell acute lymphoblastic leukemia; CR, complete response; PR, partial response; R/R, refractory/relapsed disease; MRD, minimal residual disease; ECOG, Eastern Cooperative Oncology Group; RT, radiotherapy; CRS, cytokine release syndrome; ICANS, immune effector cell-associated neurotoxicity syndrome; SCT, stem cell therapy; PB, peripheral blood.

Between September 2020 and May 2024, a total of 31 patients were screened, with 7 patients at Vitebsk Regional Clinical Cancer Center (VRCCC) and 16 at Belarusian Research Center for Pediatric Oncology, Hematology and Immunology (BRC POHI) ultimately receiving treatment between October 2020 and September 2024. Eight patients were not treated due to uncontrolled infectious complications and rapid disease progression; six of these patients died before peripheral blood could be collected for CAR T cell manufacturing, and two died prior to CAR T cell infusion (Figure 1C). The median age was 45 years (range: 38–56) at VRCCC and 13.5 years (range: 4–30) at BRC POHI. At BRC POHI, 14 patients had B-ALL, and two had Burkitt lymphoma. At VRCCC, one patient had CML (chronic myeloid leukemia) in B cell lymphoid blast crisis, two had B-ALL, one had primary mediastinal large B cell lymphoma, one had DLBCL, one had primary CNS lymphoma (PCNSL), and one had CLL (Figure 2). The median number of prior therapy lines was 3 (range: 2–6). Seven out of sixteen patients (44%) with B-ALL underwent alloSCT, while eight patients (50%) received blinatumomab prior to CD19 CAR T cell infusion. Nineteen of twenty-three patients (83%) had a T cell count exceeding 500 cells/μL. Extramedullary involvement was reported in 10 (55.5%) of 18 patients with B-ALL, with CNS involvement documented in one patient at the time of CD19 CAR T cell infusion. Most B-ALL patients exhibited M1 bone marrow status (*n* = 10), while M2 and M3 statuses were observed in four patients each. Bridge therapy between leukapheresis and lymphodepletion was utilized in seven patients—radiotherapy in two at VRCCC and chemotherapy in five at BRC POHI. Four patients with B-ALL received a second infusion of CD19 CAR T cells at BRC POHI (Figure 2).

### Characteristics of manufactured CD19 CAR T cells

**Figure 3:**
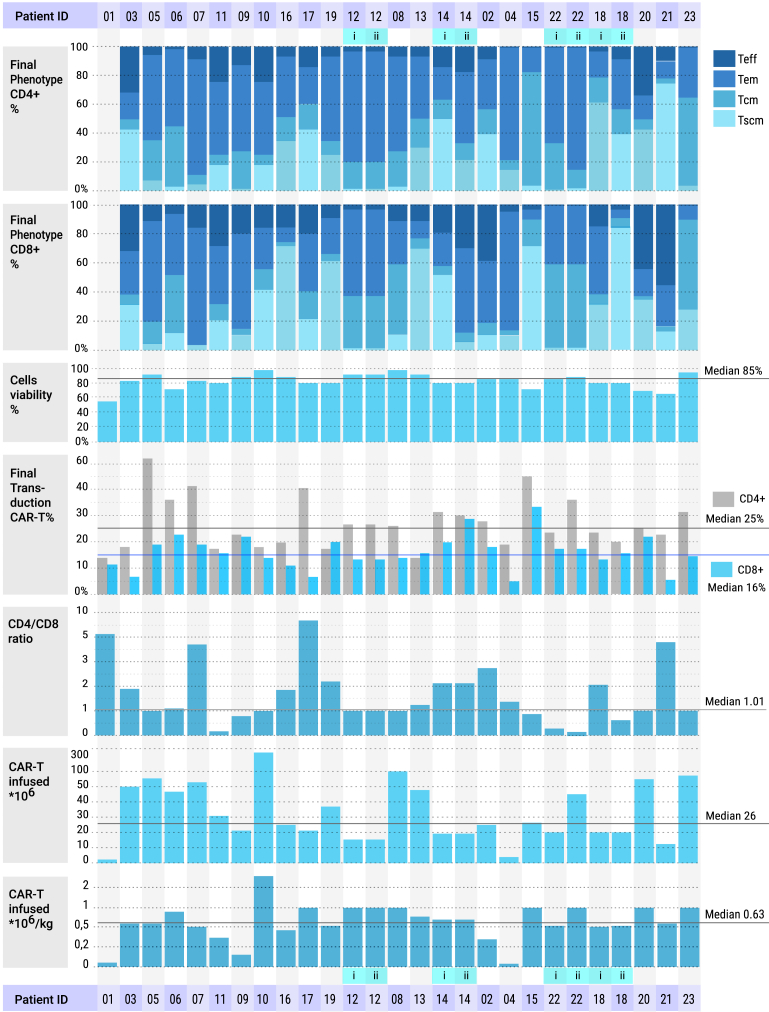
General characteristics and subpopulation analysis of the final CAR T cell product Teff, terminal effector T cells; Tem, effector memory T cells; Tcm, central memory T cells; Tscm, stem cell memory T cells.

Leukapheresis was performed in patients diagnosed with relapsed/refractory (R/R) B cell malignancies who had blood T cell counts above 150 cells/μL on the day of collection. For four patients (pt12, pt16, pt19, and pt20), T cells from haploidentical donors were used as the starting material for CAR T cell manufacturing. The average volume of the apheresis product was 117 mL (range: 70–250 mL). On average, 5 × 10^9^ (range: 0.015–64.6 × 10^9^) peripheral blood mononuclear cells (PBMCs) were isolated from the apheresis product. In seven cases, the PBMC fraction was manually isolated and cryopreserved for later use; in the remaining cases, CAR T cell production proceeded immediately. Activation and transduction of CD4^+^ and CD8^+^ lymphocytes were performed separately, followed by expansion in the presence of interleukin (IL)-7 and IL-15. The selected CD4^+^ and CD8^+^ T cells were transduced with a lentiviral vector BY19. The production process took between 7 and 31 days (median: 13 days). The resulting CAR T cell products were administered immediately to 18 patients; for five patients, the products were cryopreserved until use. The quality of the initial material varied significantly, particularly regarding the content of naive T cells and stem cell memory T cells (Tscm), with median 28.3% (range 0%–79%) for CD4^+^ T cells and median 25.8% (range 0%–82%) for CD8^+^ T cells (Figure 3). Two patients (Pt03 and Pt05) underwent immunomagnetic CD45RA selection of T cells prior to CAR T manufacturing. *In vitro* expansion over 12 days revealed a median fold increase of 13 (range: 0.24–188) for CD4^+^ cells and 11 (range: 0–195) for CD8^+^ cells.

The median cell viability was 84.5% (range: 55%–96%). Transduction efficiency was higher for CD4^+^ lymphocytes, with a median of 24.5% (range: 13%–63%), compared to CD8^+^ lymphocytes, which had a median of 16.35% (range: 6%–33%). The desired CD4:CD8 ratio in the final product was 1:1; however, due to an insufficient total dose and limited CD8^+^ cell numbers, the actual CD4:CD8 ratio varied from 0.1 to 6.9, with a median of 1.03. The median CAR T cell dose infused was 0.6 × 10^6^ cells/kg (range: 0.015–2.8 × 10^6^ cells/kg). Overall, the final CAR T cell products contained a substantial fraction of memory T cells—Tscm and central memory T cells subsets—with median proportions of 44.4% (range: 5.2%–84%) for CD4^+^ cells and 37.8% (range: 2.8%–90%) for CD8^+^ cells, confirming the retention of a memory phenotype in the manufactured products.

### Safety

**Figure 4:**
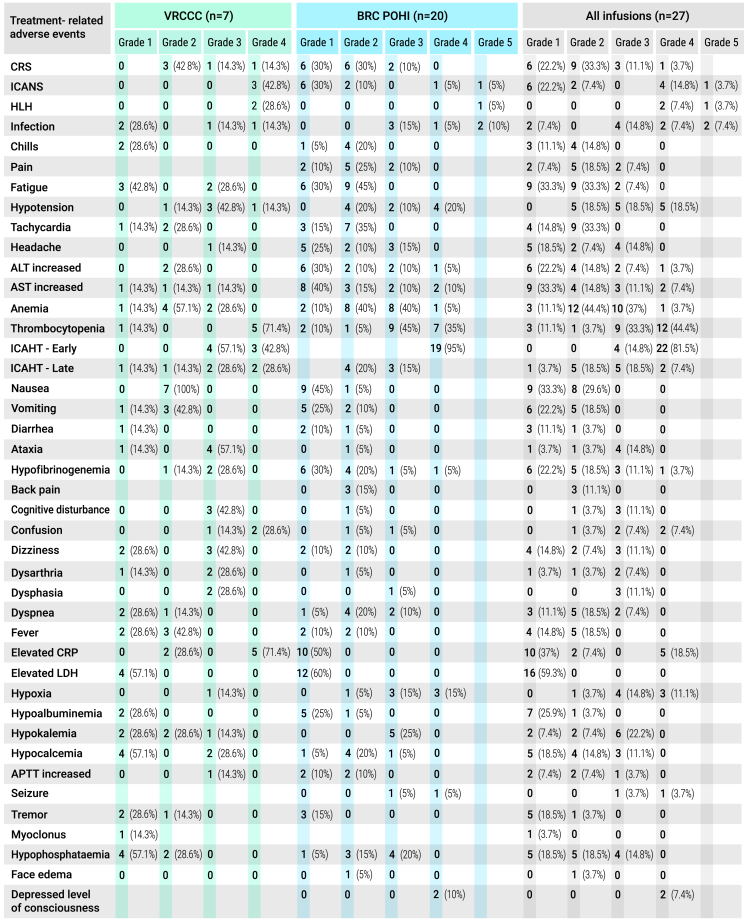
Adverse events occurred after anti-CD19 CAR T cell therapy CRS, cytokine release syndrome; ICANS, immune effector cell-associated neurotoxicity syndrome; HLH, hemophagocytic lymphohistiocytosis; ALT, alanine aminotransferase; AST, aspartate aminotransferase; ICAHT, immune effector cell-associated hematotoxicity; LDH, lactate dehydrogenase; CRP, C-reactive protein; APTT, activated partial thromboplastin time.

No acute infusion-related toxicity was observed within the first 2 h after CAR T cell infusion. Overall, safety evaluations included 27 treatment episodes, as four patients received a second infusion of CD19 CAR T cells. In the full safety cohort (*n* = 27), 92.6% of patients (*n* = 25) experienced treatment-emergent adverse events (Figure 4). Patients developed expected toxicities, CRS, immune effector cell-associated neurotoxicity syndrome (ICANS), cytopenias, and infections. CRS occurred in 71% of patients (*n* = 5) at VRCCC and 65% (*n* = 13) at BRC POHI, resulting in an overall CRS incidence of 67% (*n* = 18) across both centers, with most cases being mild. Severe CRS (≥ grade 3) was observed in 19% of patients (*n* = 5). The median time from infusion to CRS onset was 6 days (range: 1–13 days), and the median duration of CRS was 5 days (range: 1–17 days). Tocilizumab was administered after 12 CAR T infusions (52.17%), with a median 1 dose per infusion (range: 1–7 doses).

ICANS occurred in 42.8% of patients (*n* = 3) at VRCCC and 45% (*n* = 9) at BRC POHI, resulting in an overall ICANS incidence of 44% (*n* = 12) across both centers. Severe ICANS (≥ grade 3) was observed in 18.5% of patients (*n* = 5). The median time from infusion to ICANS onset was 8.5 days (range: 4–17 days), with a median duration of 3 days (range: 1–13 days). The clinical presentation of neurotoxicity was variable, ranging from mild to severe encephalopathy, focal neurological deficits, and ataxia, to more severe manifestations such as generalized seizures. All neurological symptoms and signs resolved completely over days to weeks, except for one patient who developed severe, irreversible neurological deficits 8 days after CAR T cell infusion and subsequently died. Four patients developed transient disseminated intravascular coagulation, which was successfully managed with fibrinogen concentrate, cryoprecipitate, and coagulation factor concentrates. Grade 4 hemophagocytic lymphohistiocytosis (HLH) occurred in two patients at VRCCC, and one patient at BRC POHI died due to unresponsive HLH. No GVHD was observed in any patient treated with haploidentical donor CAR T cells. Steroids were administered to 10 patients (43.4%), with a median cumulative dose of 286 mg of dexamethasone (range: 8–1145 mg) per infusion. Two patients died from severe infections in the context of CRS: one (patient 23) due to septic shock caused by pan-resistant *Klebsiella pneumoniae* and *Acinetobacter baumannii*, and another (patient 20) due to septic shock caused by multi-resistant *Pseudomonas aeruginosa*. Additional infections recorded included one case of invasive pulmonary aspergillosis, two bacterial pneumonias, two COVID-19 infections, one central line-associated bloodstream infection, one skin infection, one CMV reactivation, and one sepsis due to *Klebsiella pneumoniae*, all of which were successfully managed.

Cytopenias are a well-recognized side effect following CD19 CAR T cell therapy. Neutropenia was graded according to the EHA/EBMT consensus, while anemia and thrombocytopenia were graded using CTCAE version 4.03.^13^ In brief, most patients experienced early immune effector cell-associated hematologic toxicity (ICAHT), occurring in 81.5% (*n* = 22) of cases, predominantly of high-grade severity—grade 3 in 7.4% (*n* = 2) and grade 4 in 74.1% (*n* = 20). Late ICAHT was observed in 29.6% (*n* = 8) of patients, with grade 2 toxicity diagnosed in 5 patients (18.5%) and grade 4 in 3 patients (11.1%). Anemia was diagnosed in 26 patients (96%), with severe anemia recorded in 11 patients—grade 3 in 10 patients (37%) and grade 4 in 1 patient (3.7%). Thrombocytopenia was noted in 25 patients (92.5%), mostly of grade 3 (33%, *n* = 9) and grade 4 (44.4%, *n* = 12). By month 3, no patients exhibited severe hematological toxicity. All non-hematological adverse events were low grade and managed effectively. No cases of second primary malignancies have been documented during the observation period.

### Efficacy

**Figure 5:**
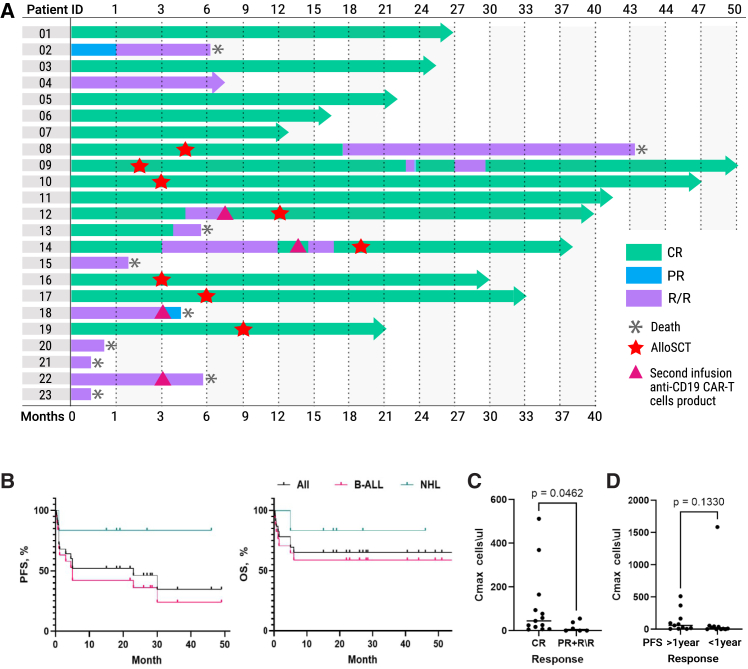
Clinical outcomes after CD19 CAR T cell therapy (A) Swimmer’s plot illustrating responses and clinical courses of patients who underwent CD19 CAR T cell therapy. (B) Progression-free survival (PFS) and overall survival (OS) stratified by disease type. CR, complete response; PR, partial response; R/R, refractory/relapsed disease; B-ALL, B cell acute lymphoblastic leukemia; NHL, non-Hodgkin lymphoma. (C) Correlation between response at 1 month and CD19 CAR T cell C_max_. The probability of achieving a response at 1-month post infusion is positively correlated with C_max_ of CD19 CAR T cells, indicating that higher CAR T cell expansion is associated with improved early response rates (CR vs. PR or R/R; *p* = 0.0462). (D) Correlation between 1-year PFS and CD19 CAR T cell C_max_ (*p* = 0.1330).

The cutoff date for the updated analysis was June 17, 2024, resulting in a median follow-up of 15.4 months. Six patients responded to therapy, yielding an ORR of 85.7% (6/7; 95% confidence interval [CI]: 42.1%–99.6%), with five CR, corresponding to a CR rate of 71.4% (5/7; 95% CI: 29.0%–96.3%) at VRCCC (Figure 5A).

At BRC POHI, 10 patients achieved CR, resulting in an overall CR rate of 76.9% (10/13 evaluable patients; 95% CI: 46.2%–95.0%). The combined ORR across both centers was 80.0% (16/20; 95% CI: 56.3%–94.3%), with a CR rate of 75.0% (15/20; 95% CI: 50.9%–91.3%) observed at the first disease assessment on day 28. Among six patients with NHL, five responded to therapy, resulting in an ORR of 83.3% (5/6; 95% CI: 35.9%–99.6%) and a CR rate of 83.3% (5/6; 95% CI: 35.9%–99.6%).

In the cohort of 14 evaluable patients with B-ALL, response assessment on day 28 following the first infusion of CD19 CAR T cells showed an ORR of 78.6% (11/14; 95% CI: 49.2%–95.3%). CR was achieved in 71.4% of B-ALL patients (10/14; 95% CI: 41.9%–91.6%), with MRD-negative remissions detected in 57.1% (8/14; 95% CI: 28.9%–82.3%) as determined by high-resolution flow cytometry. A second infusion of CD19 CAR T cells was administered to four patients, with two of them responding. Patients with lymphoma who achieved CR demonstrated very durable responses, with no relapses reported at the time of manuscript writing. Eight patients with B-ALL who responded to CD19 CAR T cell therapy proceeded to alloSCT, of whom six remain in CR at the latest follow-up. One patient (Pt03), who underwent lymphodepletion with DAC, fludarabine, and cyclophosphamide and did not proceed to alloSCT, remains in CR without any further therapy. Median progression-free survival (PFS) was 23 months, and the median OS was not reached. 12-month PFS was 83.3% for patients with NHL and 48.3% for patients with B-ALL (Figure 5B).

### Cellular kinetics of anti-CD19 CAR-T cells

**Figure 6:**
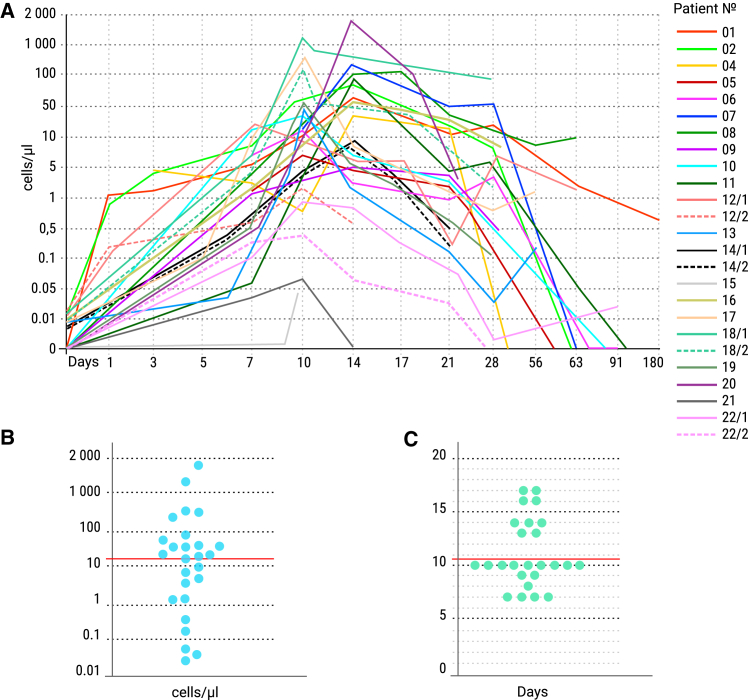
Pharmacokinetics of CD19 CAR T cells in the peripheral blood of patients (A) Expansion and persistence of CD19 CAR T cells over time. (B) Peak concentration (C_max_) of CD19 CAR T cells measured in peripheral blood, expressed as cells/μL. (C) Time to maximum concentration (T_max_) of CD19 CAR T cells in peripheral blood, expressed in days.

CAR T cell persistence was assessed in peripheral blood by flow cytometry at two or more time points after infusion in most patients. At peak expansion, CAR T cells comprised up to 48% of all viable nucleated cells (median: 11.6%; range: 1.5%–48.3%) (Figure 6A).

Regardless of the CD4:CD8 ratio in the final CAR T cell product, most patients exhibited predominant expansion of CD8 CAR T cells after infusion. All evaluable patients, except two (Pt15 and Pt21), demonstrated CD19 CAR T cell expansion in peripheral blood, with a median C_max_ of 21.7 cells/μL (range: 0.045–1,858 cells/μL) and a median T_max_ of 10 days (range: 7–17 days) (Figures 6B and 6C). A significant correlation was observed between CAR T cells C_max_ and CR attainment (*p* = 0.0462), whereas no correlation was found with 1-year PFS (*p* = 0.1330) (Figures 5C and 5D).

## Discussion

This first-in-region phase 1 study demonstrates the feasibility, safety, and preliminary efficacy of an in-house manufactured CD19 CAR T cell product (BY19) for patients with R/R B cell malignancies in Belarus. Our experience provides a clinically actionable framework for developing academic CAR T cell programs in low- and middle-income countries, where access to commercial cell therapies remains limited due to prohibitive costs, centralized logistics, and regulatory hurdles.

The BY19 product features a second-generation CAR construct with an IgG4 hinge, a CD28 costimulatory domain, and a CD3ζ signaling domain and was jointly developed by the Institute of Bioorganic Chemistry of NASB and BRC POHI, produced in an academic facility under GMP-adapted procedures at BRC POHI. The entire production cycle, including leukapheresis, transduction, expansion, and quality control, was completed in a median of 13 days. Both fresh and cryopreserved products were successfully transported from the manufacturing facility at BRC POHI to the clinical site at VRCCC, confirming the logistical and functional viability of academic manufacturing and inter-site transport within a national network.

Conducting scientific research and clinical testing of our CD19 CAR T cell product occurred during a period when extensive data from various phase 1/2 clinical trials exploring different anti-CD19 CAR designs were available. Among 39 studies that tested at least two dose levels of CAR T cells, an association between dose administered and ORR or CR was observed in only 13 (33%) studies, raising questions about a strict dose-response relationship.^14^ Notably, in these studies, the starting dose was comparatively lower (<30 million cells), whereas in studies showing no correlation between dose and disease response, the starting dose or dose level 1 exceeded 50 million cells. Since the minimum effective dose often occurs well before the maximum tolerated dose in traditional phase 1 trials for hematological malignancies, there is a strong rationale to identify an optimal dose window—one that maximizes efficacy without increasing the risk of toxicity and adverse events.^15^ Additionally, managing severe CRS and ICANS may lead to higher cumulative corticosteroid doses, which have been associated with significantly shorter PFS and OS.^16^ Considering that most CD19 CAR T cell studies achieved optimal clinical efficacy (greater than 70% ORR) at doses between 50 and 100 million cells, we selected these doses for our CD19 CAR T cell study in adult patients.

Moreover, we incorporated DAC into the lymphodepletion regimen for adult patients prior to CAR T cell infusion. DAC is a nucleoside analog whose phosphate group covalently binds to DNA methyltransferase, inhibiting its activity. As a result, DAC functions as a hypomethylating agent by promoting DNA demethylation.^17^ Preclinical evidence demonstrates that preincubation of CD19^+^ lymphoma cell lines with DAC significantly enhances the cytotoxic capacity of anti-CD19 CAR T cells.^18^ One direct mechanism involves DAC-mediated upregulation of CD19 expression on lymphoma cells, while indirect *in vivo* effects may include disruption of the immunosuppressive tumor microenvironment.^19^^,^^20^^,^^21^ In a small study, incorporating DAC into lymphodepletion significantly improved response durability, resulting in longer PFS and OS in patients with B-ALL receiving CD19/CD22 CAR T cells.^22^

An additional advantage of DAC is its ability to penetrate the CNS, which could be highly valuable for improving outcomes in CNS lymphoma treated with CAR T cell therapy, given its immunomodulatory activity discussed earlier.^23^ The successful outcome of our PCNSL patient, who received DAC-based lymphodepletion followed by anti-CD19 CAR T cell therapy, supports further investigation into this approach. The toxicities associated with DAC-based lymphodepletion combined with CAR T cell therapy were manageable, with only two cases of CRS among seven patients and three cases of ICANS.

Interestingly, our CD19 CAR T cells combined with DAC-based lymphodepletion demonstrated high efficacy against multiple types of NHL, comparing favorably to the response rates observed with FDA-approved products such as axicabtagene ciloleucel, lisocabtagene maraleucel, and tisagenlecleucel.^24^^,^^25^ The remission rates, EFS, and OS for patients with B-ALL in our study align with the lower-end results reported for other CD19 CAR T cell products.^9^^,^^26^^,^^27^ These outcomes may be attributed to a heavily pretreated patient population, including prior therapies with blinatumomab, high tumor burden, extramedullary disease such as CNS involvement, and colonization with multidrug-resistant Gram-negative bacteria, which contributed to two sepsis-related deaths. This underscores that infections remain a significant challenge in highly pretreated hematological patients undergoing CAR T cell therapy and could contribute to excessive mortality rate.^26^ Our observations suggest that patients with higher C_max_ tend to achieve higher response rates. However, this study has limitations, including the small sample size and heterogeneity of the patient population. Consequently, we cannot draw definitive conclusions regarding safety and efficacy or establish conclusive correlative associations. Nonetheless, DAC-based lymphodepletion appears promising and warrants further investigation. To address these issues more robustly, ongoing recruitment across different disease cohorts is essential to gather sufficient data for definitive assessments of safety and efficacy across various types of lymphoid neoplasms.

This phase 1 study establishes the feasibility and clinical potential of decentralized, academic CAR T cell therapy in a resource-limited setting. The BY19 product, manufactured locally and infused at national cancer centers in Belarus, produced encouraging clinical responses with a manageable safety profile. The integration of DAC into lymphodepletion may enhance antitumor activity and deserves further exploration, particularly in CNS lymphoma.

An important consideration for academic CAR T cell programs in resource-limited settings is economic feasibility. The estimated total manufacturing cost for one BY19 product is approximately 25,000 USD, while the clinical implementation and patient management costs are typically in the range of 20,000–30,000 USD per treatment episode. In contrast, the total cost of commercial CAR T cell products—including manufacturing, logistics, and hospital care—exceeds 350,000–500,000 USD per patient.

Importantly, the BY19 manufacturing process does not rely on automated closed systems such as the CliniMACS Prodigy, which require proprietary, high-cost disposable kits and reagents.^28^ Instead, we implemented a semi-manual, GMP-adapted workflow using standard cell culture equipment and open but well-controlled procedures. This approach significantly reduced production expenses while maintaining full compliance with national regulatory and biosafety standards.

The comparatively low production and treatment costs of BY19 were achieved through in-country manufacturing, use of standard hospital infrastructure, and streamlined regulatory pathways. This model demonstrates that safe and effective CAR T cell therapy can be developed and delivered sustainably within middle-income healthcare systems, providing a viable blueprint for other developing countries seeking to establish local cell therapy capacity.

Our experience demonstrates that safe, effective, and scalable CAR T cell therapy can be developed and delivered outside of high-income academic hubs. This model offers a compelling blueprint for improving access to transformative cell therapies globally and underscores the importance of continued investment in locally adaptable immunotherapy.

## Materials and methods

### Study design and participants

Adult and pediatric patients were enrolled in a single-arm, an open-label, non-randomized, parallel phase 1 clinical trial (ClinicalTrials.gov identifiers: NCT05333302) conducted at two independent centers: VRCCC and the BRC POHI. The study was approved by the Local Ethical Committees and Institutional Review Boards of BRC POHI and VRCCC and was conducted in accordance with the Declaration of Helsinki and International Conference on Harmonization guidelines for Good Clinical Practice. All patients provided written informed consent. Patients with R/R CD19^+^ lymphoid neoplasms received autologous or allogeneic CD19 CAR T cells manufactured at BRC POHI, utilizing clinical-grade lentiviral vectors and following standard operating procedures for cell production. Bridging chemotherapy was permitted prior to lymphodepletion. Lymphodepletion protocols adopted at two clinical centers are depicted in Figure 1B.

### CAR expression cassettes

The BY19 CAR cassette encodes a scFv derived from the CD19 antibody (FMC63), preceded by the CSF2R signal peptide and linked to the short hinge (12 amino acids) derived from human IgG4 domain. This is followed by the transmembrane domain of CD28, the 4-1BB co-stimulatory domain, and the CD3ζ (zeta) signaling chain (Table S1).

For comparison, a CD28-based CD19 CAR (28Z) was designed to mimic the axicabtagene ciloleucel configuration. This construct contains the FMC63 scFv linked to the CD28 hinge, transmembrane, and co-stimulatory domains, followed by the CD3ζ signaling chain. Both CAR constructs include a membrane-anchored truncated epidermal growth factor receptor (tEGFR) domain separated from the CD3ζ chain by a GSG linker and a P2A self-cleaving peptide sequence.

### Production of recombinant lentiviral particles

Recombinant lentiviral particles were produced by transient transfection of 293T packaging cells with the second-generation transfer vector pWPXL (encoding the respective expression cassettes), the packaging plasmid pCMV-dR8.91, and the envelope plasmid pMD2.G, using linear polyethylenimine (Serochem, Prime-AQ100-100ML). 48 h post transfection, virus-containing supernatants were collected and clarified by centrifugation, filtered through 0.45 μm syringe filters, and concentrated by low-speed centrifugation (3,000 × g, 4°C, 24 h). Viral pellets were resuspended in ImmunoCult-XF T cell Expansion Medium (STEMCELL Technologies, Inc., 10981) and stored at −80°C. Functional titers were determined by transducing 293T cells with serial dilutions of the viral preparation, followed by flow cytometric analysis of transduction efficiency.

### Generation of CAR T cells for clinical use

PBMCs were isolated from leukapheresis products of enrolled patients by density gradient centrifugation using ROTI-Sep 1077 human solution (Carl Roth GmbH + Co. KG, 0642.2). In seven cases, the PBMC fraction was cryopreserved; in the remaining cases, cells were processed immediately for CAR T cell manufacture. CD4^+^ and CD8^+^ T lymphocytes were manually isolated using Dynabeads CD4 and CD8 Positive Isolation Kits (Thermo Fisher Scientific, 11331D and 11333D). Two patients (Pt03 and Pt05) underwent immunomagnetic selection of naive T cells EasySep Human Naive CD4/8^+^ T cell Isolation Kits (STEMCELL Technologies, Inc., 19555 and 19258) for CAR T production.

Following enrichment, CD4^+^ and CD8^+^ T cells were separately stimulated with CTS Dynabeads CD3/CD28 (Thermo Fisher Scientific, 40203D). 48 h post activation, T cells were genetically modified with pre-generated recombinant lentiviral particles encoding BY19 expression cassette via spinoculation on RetroNectin (Takara Bio, Inc., T202)-coated plates at a multiplicity of infection of 5. Activation, transduction, and subsequent expansion were performed in ImmunoCult-XF T cell Expansion Medium (STEMCELL Technologies, Inc., 10981) supplemented with recombinant human IL-7 (Miltenyi Biotec, 170-076-111) and IL-15 (Miltenyi Biotec, 170-076-114) each at a final concentration of 10 ng/mL.

In-process quality control included the evaluation of T cell selection purity, transduction efficiency, and culture viability. The purity of immunomagnetically selected T cells was assessed by flow cytometry (acceptance criterion: >98% CD3^+^ cells). Genetic modification efficiency was evaluated on days +3 and +6 post transduction by flow cytometry (acceptance criterion: overall transduction efficiency >10%). Cell viability was assessed every 72 h throughout the manufacturing process by flow cytometry (acceptance criterion: >70%). Visual inspection of the cultures was performed daily to monitor medium clarity and detect potential signs of microbial contamination.

Final product release testing included sterility assessment using the BACT/ALERT 3D system (bioMérieux; acceptance criterion: no microbial growth after 7 days), mycoplasma testing by PCR (acceptance criterion: no specific amplification), and endotoxin testing using the PYROGENT-5000 Kinetic Turbidimetric LAL Assay (Promega; acceptance criterion: <0.5 EU/mL).^29^ Replication-competent lentivirus was assessed by qPCR (acceptance criterion: no specific amplification).^30^ Final product purity was confirmed by flow cytometry (acceptance criterion: absence of contaminating cell populations), while transduction efficiency (acceptance criterion: >10%) and viability (acceptance criterion: >70%) were also verified by flow cytometric analysis.

### Flow cytometry

Flow cytometry acquisition was performed with a DxFlex flow cytometer (Beckman Coulter, C78505). Data analysis was performed using CytExpert software (Beckman Coulter, CytExpert for DxFLEX 2.0).

CARs identification on the surface of CAR T cells was performed by biotinylated recombinant CD19 protein (in-house production) and streptavidin-APC conjugate (BioLegend, 405207). tEGFR reporter was identified by Alexa Fluor 488-conjugated biosimilar of cetuximab (R&D Systems, FAB9577G-100).

The phenotypic composition and quantity of T lymphocytes in the patient’s blood before apheresis, PBMCs, and final CAR T product were determined by staining the cells with antibodies CD45 (Beckman Coulter. c.J33), CD4 (Invitrogen, c.RPA-T4), CD8 (Invitrogen, c.RPA-T8), CD3 (Beckman Coulter, c.UCHTI), CD45RO (Invitrogen, c.HI100), and CCR7 (Miltenyi Biotec, c.REA546). CAR T cell persistence after infusion in blood, bone marrow, and cerebrospinal fluid was monitored using antibodies CD45 (Beckman Coulter. c.J33), CD4 (Invitrogen, c.RPA-T4), CD8 (Invitrogen, c.RPA-T8), CD3 (Beckman Coulter, c.UCHTI), and human tEGFR cetuximab biosimilar (Invitrogen c.Me183). DAPI (Invitrogen, 62248) was used as a viability dye.

### Statistical analysis

All *in vitro* assays were performed in triplicates. Data are presented as mean ± standard deviation. Group comparisons were performed using ordinary one-way ANOVA, with differences considered statistically significant at a *p* value <0.05. Statistical analyses and data visualization were conducted using GraphPad Prism software, version 8 (GraphPad Software, San Diego, CA, USA).

Standard descriptive statistics, including median/range, percentage, and response rate, were reported for key variables for clinical result assessment. Directional associations between categorical variables with three levels and various predictors were evaluated using univariate proportional odds logistic regression. A *p* value <0.05 was considered statistically significant.

Kaplan-Meier curves were generated for OS and PFS. The OS and PFS curves included data from all infused patients. Log rank tests were performed to assess significance between these groups.

For the engraftment analysis, peak engraftment (expressed as a percentage or absolute number of total T cells) and time-averaged engraftment (measured as the area under the curve) from infusion to day +90 were calculated for each patient. These values were then grouped by risk factors as outlined in this section, and differences were evaluated using a two-sided Wilcoxon-Mann-Whitney test (for comparisons between two groups) or a Kruskal-Wallis test (for comparisons involving three or more groups).

### Endpoints and study procedures

The primary endpoint was safety. Secondary endpoints included ORR, CR, PFS, and OS, as well as the expansion and persistence of CAR T cells. CRS and ICANS were graded according to the consensus criteria of the American Society for Transplantation and Cellular Therapy.^31^ HLH was graded based on Shah N.N. criteria.^32^ ICAHT grading followed the EHA/EBMT consensus guidelines.^33^ All other toxicities were graded using the National Cancer Institute’s Common Terminology Criteria for Adverse Events, version 4.0.

Response assessment for lymphoma and B-ALL with extramedullary involvement was performed according to the Lugano classification 2014, utilizing FDG PET-computed tomography (CT) or CT scans at +28 days post treatment and subsequently every 3 months.^34^ Brain MRI was employed to evaluate CNS involvement. Responses in B-ALL were graded according to standard criteria, while CLL/small lymphocytic lymphoma responses were assessed based on the iwCLL 2018 guidelines.^35^

## Data and code availability

The main data supporting the results in this study are available within the paper and the supplement information. All data generated in this study can be obtained from the corresponding authors upon reasonable request.

## Acknowledgments

The work was supported by the State Committee on Science and Technology of Belarus (agreement no. 201739). We sincerely appreciate the contributions of Professor Olga Aleinikova and Professor Natalya Konoplya in advancing CAR T cell therapy from the preclinical stage to clinical investigation, as well as for their mentorship. We also extend our gratitude to the physicians, nurses, and staff at the BRC POHI and VRCCC laboratories for their dedicated routine work.

## Author contributions

Conceptualization, A. Meleshko, A. Migas, D.D., and M.K.; investigation, all authors; writing – original draft, M.K. and D.D.; writing – review and editing, A. Meleshko and A. Migas; funding acquisition, A. Meleshko and D.D. All authors have read and agreed to the published version of the manuscript.

## Declaration of interests

The authors declare no competing interests.

## Supplemental information

Document S1. Figures S1 and S2 and Table S1

Document S2. Article plus supplemental information
