# Supplementary material for: CD19 CAR T cell therapy BY19 for pediatric and adult patients with relapsed or refractory B cell neoplasms in Belarus: Phase 1 trial
Source: Mol Ther Oncol. 2025 Nov 1;33(4):201081. doi: 10.1016/j.omton.2025.201081 (PMC12664381; doi:10.1016/j.omton.2025.201081)
Supplement: Document S1. Figures S1 and S2 and Table S1 [file mmc1.pdf]

OMTON, Volume *33*

Supplemental information

CD19 CAR T cell therapy BY19 for pediatric and adult patients with relapsed or refractory B cell neoplasms in Belarus: Phase 1 trial

Mikalai Katsin, Dmitri Dormeshkin, Alexandr Migas, Olga Karas, Tatsiana Shman, Yuliya Serada, Yauheniya Khalankova, Hanna Klych, Dzmitry Lutskovich, Alena Lukoika, and Alexander Meleshko

SUPPLEMENTAL MATERIAL

Cell lines and general cell culture

B-cell malignant cell lines Raji (ATCC CCL-86) and CII (DSMZ ACC-773), chronic myelogenous leukemia cell line K562 (ATCC CCL-242), embryonic kidney cell line 293T (ATCC CRL-3216) and T-cell leukemia cell line Jurkat E6.1 (ATCC TIB-152) were originally purchased from the American Type Culture Collection (ATCC) or the Deutsche Sammlung von Mikroorganismen und Zellkulturen (DSMZ). The reporter cell lines Jurkat_NFAT.GFP and Jurkat_NFkB.GFP, derivatives of the Jurkat E6.1 line, carry reporter constructs that enable expression of green fluorescent protein (GFP) in response to activation of the respective signaling pathways. These lines were generated in-house as previously described [1]. The cell lines K562, Raji, and CII were stably transduced to express the red fluorescent protein iRFP713, which served as a unique identifier for flow cytometric analysis in mixed cultures. All cell lines were maintained in accordance with the respective collection guidelines.

Generation of CAR T-cells and CAR-Expressing Jurkat Reporter Lines for in vitro tests

Primary $CD4^+$ and $CD8^+$ T-lymphocytes were isolated from the peripheral blood of a healthy donor by immunomagnetic selection (Miltenyi Biotec, 130-096-535; 130-042-201). T-cells were activated using magnetic beads (Miltenyi Biotec, 130-091-441). 48 h post-activation, T-cells were transduced with pre-generated recombinant lentiviral particles encoding BY19 or 28Z expression cassettes via spinoculation in the presence of Vectofusin-1 transduction enhancer (Miltenyi Biotec, 130-111-163) at MOI of 5. Cells were expanded for 12–14 days in RPMI-1640 medium (Capricorn, RPMI-HA) supplemented with 10% FBS (Capricorn, FBS-HI-12A), recombinant human interleukin-7 (Miltenyi Biotec, 130-095-362), and interleukin-15 (Miltenyi Biotec, 130-095-764), each at a final concentration of 10 ng/mL.

Genetic modification of the parental Jurkat E6.1 cell line and its reporter derivatives, Jurkat_NFAT.GFP and Jurkat_NFkB.GFP, was performed using pre-generated recombinant lentiviral particles encoding BY19 or 28Z expression cassettes at MOI of 0.2.

In vitro assessment of CAR Functionality

To assess CAR surface expression, Jurkat E6.1 cells were transduced with recombinant lentiviral particles carrying the corresponding expression cassettes. Genetically modified cells were stained with recombinant CD19 protein to detect the FMC63-based scFv and with a cetuximab biosimilar to detect the tEGFR reporter. CAR expression was normalized by calculating the ratio of the FMC63 scFv mean fluorescence intensity (MFI) to that of tEGFR.

To evaluate the functional activity of the CAR intracellular signaling domains, reporter cell lines Jurkat_NFAT.GFP and Jurkat_NFkB.GFP expressing the analyzed CARs were co-cultured with target cells (K562, Raji, CII) at an effector-to-target (E:T) ratio of 1:1 for 24 h. GFP reporter expression was measured by flow cytometry at 0, 4, and 24 h.

In vitro assessment of Primary CAR T-cells Functional Activity

Primary CAR T cells, generated from healthy donor material, were incubated for 48 h in complete medium without adding cytokines prior to assays initiation. Cells were then washed and mixed with non-transduced T cells to adjust the final CAR-positive fraction to 25%.

For the 24 h co-cultivation assay, effector cells were mixed with target cells (K562, Raji, CII) at effector-to-target (E:T) ratios of 1:1, and incubated for 24 h prior to flow cytometric analysis. The fold expansion of target cells was determined by dividing the number of live target cells detected after 24 h of co-cultivation with CAR T cells by the number of input target cells at assay initiation.

For the rechallenge assay, effector cells were co-cultured with target cells (Raji, CII) at an E:T ratio of 1:1. Every 48–72 h, cell content was analyzed by flow cytometry and fresh live target

cells were added in the same quantity as the initial input. A total of five restimulation rounds were performed.

Target cell content was determined by flow cytometry based on iRFP713 expression, and CAR T cell content was determined based on tEGFR expression.

CAR-T culture supernatants were assessed for IFNγ production after 24 h of co-cultivation with target cells using an ELISA kit (ElabScience, E-UNEL-H0069), following the manufacturer's instructions.

Figure S1

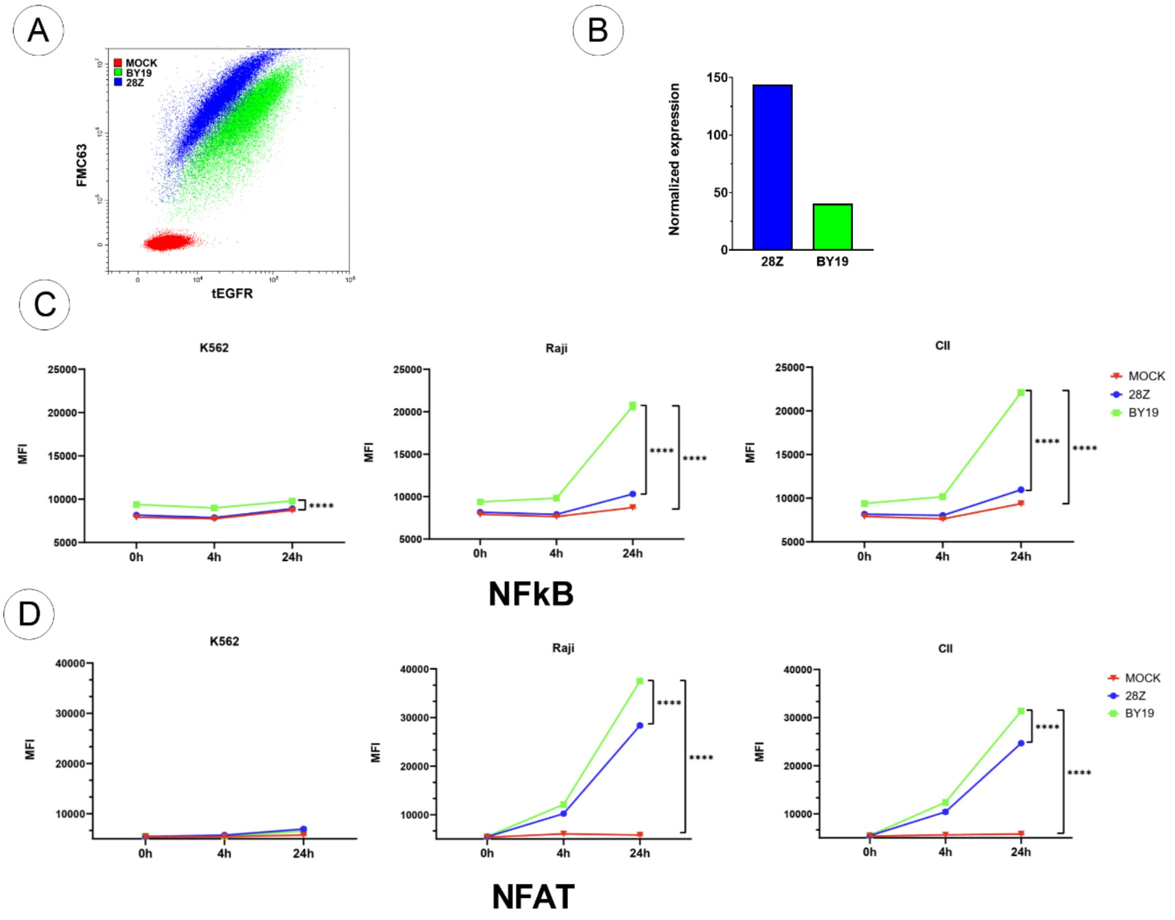

<u>Figure S1</u>: In vitro assessment of CAR Functionality. (A) CAR expression of transduced T-cells. Extracellular modules of BY19 and 28Z were directly stained with biotin-labeled CD19 protein and tEGFR reporter was stained with cetuximab biosimilar. MOCK - untransduced cells; **(B)** CAR expression normalized to tEGFR. BY19 and 28Z MFI levels were normalized to the MFI levels of tEGFR reporter protein expression; **(C, D)** CAR mediated activation of NFkB and NFAT signaling cascades. EGFP expression kinetics of stimulated BY19 and 28Z CAR Jurkat reporter cells. Data represent MFI ± SD. MOCK - untransduced cells.

Figure S2

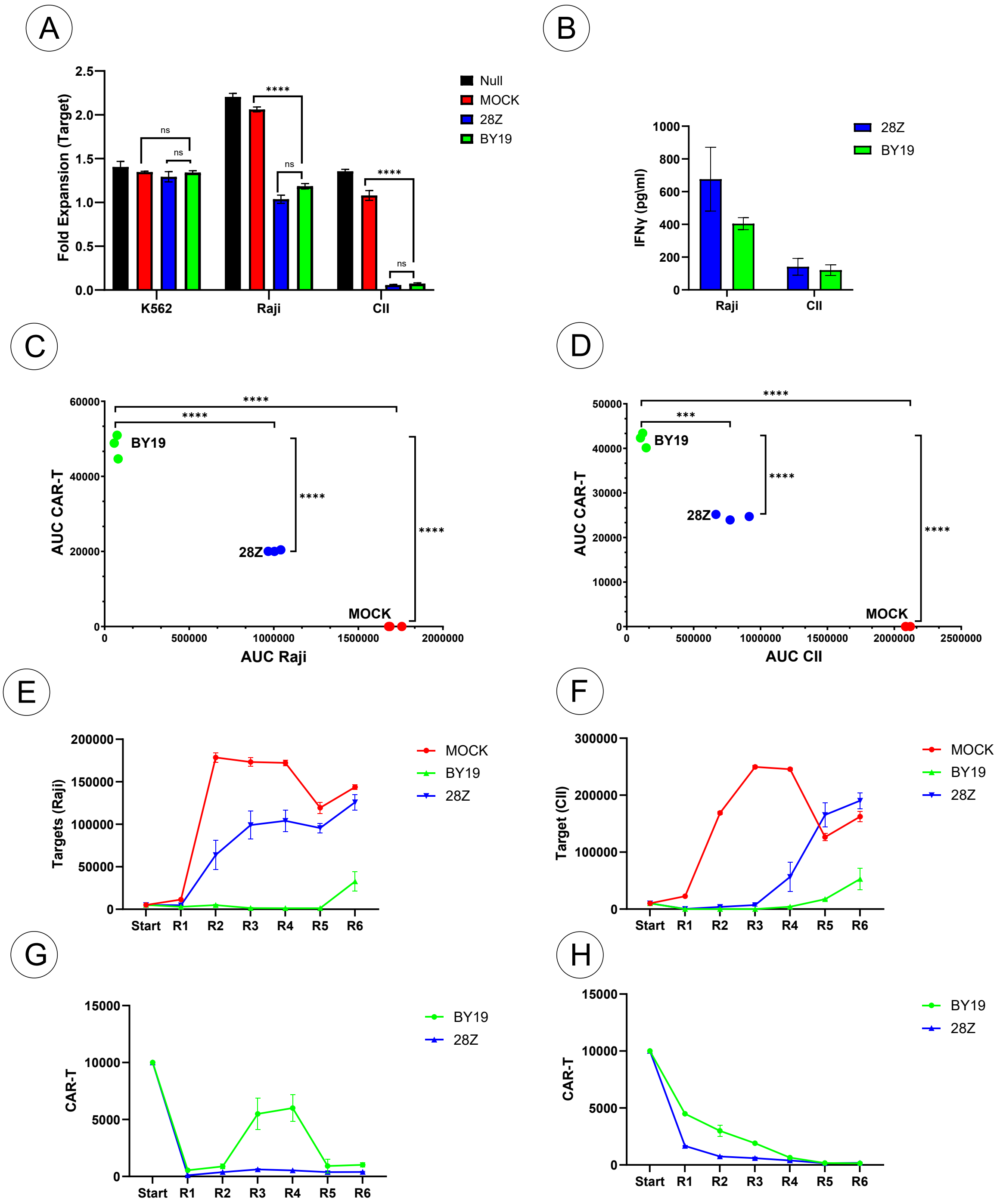

<u>Figure S2</u>*: In vitro* cytotoxicity assessment of BY19 CAR T-cells compared to 28Z CAR T-cells. (A) CAR mediated cytotoxic activity of T-cells in 24h co-cultivation assay. Fold expansion of target cells in short term co-cultivation assay with BY19 and 28Z CAR T-cells at E:T ratio 1:1. Data represent Mean ± SD. MOCK - co-culture of target cells with untransduced

T-cells. Null - culture of target cells alone; **(B)** ELISA-based quantification of IFNγ production. Supernatants after 24h co-culture of engineered CAR T-cells with Raji or CII target cells. Data represent means ± SD; **(C, D)** Control of Raji and CII cells and proliferation of CAR T cells in rechallenge assay. AUC of target and CAR T cells in five rounds of rechallenge assay. MOCK - co-culture of target cells with untransduced T-cells; **(E, F)** Target cells (Raji and CII) expansion upon each round of repetitive stimulation; **(G, H)** CAR-T expansion upon rechallenge stimulations with Raji and CII respectively.

Table S1.: Amino Acid Sequences and domain compositions of employed CAR designs.

Name	Amino Acids Sequence
BY19	MLLLVTSLLLCELPHPAFLLIP – **leader peptide**<br>DIQMTQTTSSLSASLGDRVTISCRASQDISKYLNWYQQKPDGTVKLLIYHTSRLHSGV<br>PSRFSGSGSGTDYSLTISNLEQEDIATYFCQQGNTLPYTFGGGTKLEITGSTSGSGKPG<br>SGEGSTKGEVKLQESGPGLVAPSQSLSVTCTVSGVSLPDYGVSWIRQPPRKGLEWLG<br>VIWGSETTYYNSALKSRLTIIKDNSKSQVFLKMNSLQTDDTAIYYCAKHYYYGGSYA<br>MDYWGQGTSVTVSSAAA – **FMC63 scFv;**<br>ESKYGPPCPPCP – **IgG4 hinge ;**<br>FWVLVVVGGVLACYSLLVTVAFIIFWV – **CD28 transmembrane**;<br>KRGRKKLLYIFKQPFMRPVQTTQEEDGCSCRFPEEEEGGCEL – **4-1BB co-stimulatory domain;**<br>RVKFSRSADAPAYQQGQNQLYNELNLGRREEYDVLDKRRGRDPEMGGKPRRKNPQ<br>EGLYNELQKDKMAEAYSEIGMKGERRRGKGHDGLYQGLSTATKDTYDALHMQALP<br>PR **– CD3z signaling domain;**<br>GSGATNFSLLKQAGDVEENPGP – **linker and P2A peptide;**<br>MLLLVTSLLLCELPHPAFLLIP - **leader peptide;**<br>RKVCNGIGIGEFKDSLSINATNIKHFKNCTSISGDLHILPVAFRGDSFTHTPPLDPQELD<br>ILKTVKEITGFLLIQAWPENRTDLHAFENLEIIRGRTKQHGQFSLAVVSLNITSLGLRSL<br>KEISDGDVIISGNKNLCYANTINWKKLFGTSGQKTKIISNRGENSCKATGQVCHALCS<br>PEGCWGPEPRDCVSCRNVSRGRECVDKCNLLEGEPREFVENSECIQCHPECLPQAMN<br>ITCTGRGPDNCIQCAHYIDGPHCVKTCPAGVMGENNTLVWKYADAGHVCHLCHPN<br>CTYGCTGPGLEGCPTNGPKIPSIATGMVGALLLLLVVALGIGLFM **– truncated human EGFR.**
28Z	MLLLVTSLLLCELPHPAFLLIP – **leader peptide**;<br>DIQMTQTTSSLSASLGDRVTISCRASQDISKYLNWYQQKPDGTVKLLIYHTSRLHSGV<br>PSRFSGSGSGTDYSLTISNLEQEDIATYFCQQGNTLPYTFGGGTKLEITGSTSGSGKPG<br>SGEGSTKGEVKLQESGPGLVAPSQSLSVTCTVSGVSLPDYGVSWIRQPPRKGLEWLG<br>VIWGSETTYYNSALKSRLTIIKDNSKSQVFLKMNSLQTDDTAIYYCAKHYYYGGSYA<br>MDYWGQGTSVTVSSAAA – **FMC63 scFv;**<br>IEVMYPPPYLDNEKSNGTIIHVKGKHLCPSPLFPGPSKP – **CD28 hinge;**<br>FWVLVVVGGVLACYSLLVTVAFIIFWV – **CD28 transmembrane**;<br>RSKRSRLLHSDYMNMTPRRPGPTRKHYQPYAPPRDFAAYRS – **CD28 co-stimulatory domain;**<br>RVKFSRSADAPAYQQGQNQLYNELNLGRREEYDVLDKRRGRDPEMGGKPRRKNPQ<br>EGLYNELQKDKMAEAYSEIGMKGERRRGKGHDGLYQGLSTATKDTYDALHMQALP<br>PR **– CD3z signaling domain;**<br>GSGATNFSLLKQAGDVEENPGP – **linker and P2A peptide;**<br>MLLLVTSLLLCELPHPAFLLIP - **leader peptide;**<br>RKVCNGIGIGEFKDSLSINATNIKHFKNCTSISGDLHILPVAFRGDSFTHTPPLDPQELD<br>ILKTVKEITGFLLIQAWPENRTDLHAFENLEIIRGRTKQHGQFSLAVVSLNITSLGLRSL<br>KEISDGDVIISGNKNLCYANTINWKKLFGTSGQKTKIISNRGENSCKATGQVCHALCS<br>PEGCWGPEPRDCVSCRNVSRGRECVDKCNLLEGEPREFVENSECIQCHPECLPQAMN<br>ITCTGRGPDNCIQCAHYIDGPHCVKTCPAGVMGENNTLVWKYADAGHVCHLCHPN<br>CTYGCTGPGLEGCPTNGPKIPSIATGMVGALLLLLVVALGIGLFM **– truncated human EGFR.**

References for supplemental methods

1. Jutz, S. *et al.* Assessment of costimulation and coinhibition in a triple parameter T cell reporter line: Simultaneous measurement of NF-κB, NFAT and AP-1. *J Immunol Methods* **430**, 10–20 (2016).
